# OA03 Idiopathic recurrent pericarditis treated with anakinra: an autoinflammatory phenomenon?

**DOI:** 10.1093/rap/rkae117.003

**Published:** 2024-11-05

**Authors:** Nicola Heyer, Arvind Kaul

**Affiliations:** St. George’s University Hospital, London, United Kingdom; St. George’s University Hospital, London, United Kingdom

## Abstract

**Introduction:**

Historically, idiopathic recurrent pericarditis (IRP) has been considered challenging to treat, with underlying pathological mechanisms being incompletely understood. Over the last few years, there has been a growing body of evidence to support IRP as an autoinflammatory disease. Consequentially, the use of interleukin-1 receptor antagonists in refractory disease is becoming increasingly common. Our case describes a patient with three distinct presentations of severe pericarditis associated with pleural effusions and systemic inflammation, despite treatment with high-dose NSAIDs and colchicine. Following initiation of anakinra, he had complete sustained symptomatic, biochemical and radiographical resolution of disease, with no further episodes.

**Case description:**

A 40-year-old Caucasian previously fit and well male presented to his local hospital with two weeks of pericarditic chest pain despite ibuprofen use. He was found to have a large pericardial effusion requiring pericardiocentesis, suspected to be secondary to viral pericarditis. On discharge his symptoms and effusion had resolved.

Two months later, his pericarditic chest pain recurred with general malaise and pyrexia. An episode of postural syncope precipitated local admission. He had no joint pain, rashes, or other symptoms or signs of connective tissue disease. He had no significant family, travel or occupational history. A CTPA showed recurrent pericardial effusion with a left-sided pleural effusion, prompting transfer to our centre for pericardiocentesis.

Blood tests revealed raised inflammatory markers (CRP 302 mg/L; WCC 14.2x10*9/L), but were otherwise unremarkable including troponin. An ECG showed sinus tachycardia only. An echocardiogram confirmed recurrence. Pericardiocentesis was attempted but unsuccessful.

Pericardial fluid from his first admission revealed scanty white cells, but no growth on bacterial or tuberculosis cultures. Pleural fluid aspiration revealed an exudative effusion. Extensive infection screening was negative. He was treated with antibiotics, high-dose aspirin and colchicine, with clinical improvement, normalisation of inflammatory markers and resolution of his pericardial effusion on repeat echocardiogram.

Four weeks later, he presented with a further collapse, recurrent chest pain and fevers. His inflammatory markers were raised (CRP 227 mg/L) and a repeat echocardiogram showed a bright, thickened pericardium with small-volume effusion. Immunology, including ANA, ANCA, rheumatoid factor, anti-CCP, anticardiolipin antibodies and serum ACE were all negative. Serum ferritin was mildly raised (653ug/L).

A diagnosis of IRP was made. Anakinra was commenced with excellent clinical response and resolution of all changes on echocardiogram. An attempt to switch to alternative maintenance therapy was not tolerated. Anakinra was continued through the national centre. He remains well with no further episodes of pericarditis.

**Discussion:**

Recurrent pericarditis is defined as a further episode of pericarditis occurring after a symptom-free interval of at least 4 weeks. Recurrence occurs in 15 to 30% of cases, with the majority being idiopathic. An immune-mediated pathogenesis has long been postulated with treatment centred around anti-inflammatory medications. While the exact disease mechanism remains elusive, recently IRP has been increasingly conceptualised as an autoinflammatory phenomenon.

Autoinflammatory disease encompasses a group of conditions driven by innate immune system overactivity with no identifiable extrinsic trigger, autoreactive antibodies or antigen-specific T-cells. The prototypical autoinflammatory conditions are monogenic disorders characterised by recurrent spontaneous episodes of systemic inflammation underpinned by disruption of interleukin-1 signalling. There is accumulating evidence that autoinflammatory and autoimmune disease form a spectrum, with monogenic disorders sitting at the extremes. Conditions, including gout, psoriasis, and IRP, are being reconsidered as predominantly autoinflammatory disorders.

One UK study compared IRP patients to those with monogenic autoinflammatory disorders. They found that IRP most frequently manifests with evidence of systemic inflammation and extrapericardial effusions, as illustrated here. Comparatively, pericarditis was a feature of monogenic disease but was uncommon, and never the initial complaint. Genetic analysis of IRP patients revealed an increased frequency of rare deleterious variants of four monogenic autoinflammatory disease genes involved in interleukin-1β processing. These clinical and genetic bridges strengthen the hypothesis that IRP is an autoinflammatory disease.

Identification of effective IRP treatments has enabled understanding of an autoinflammatory mechanism and vice versa. The effectiveness of colchicine has been used as evidence of IRP as an interleukin-1 driven process. The poor outcomes associated with corticosteroid dependence in refractory IRP has led to exploration of targeted interleukin-1 therapy. The AIRTRIP trial demonstrated a significant reduction in pericarditis recurrence with anakinra versus standard care in IRP. Similarly, this case shows rapid disease control with anakinra, avoiding corticosteroid use.

**Key learning points:**

• Autoinflammatory and autoimmune disease are increasingly being considered on a spectrum, with prototypical monogenic autoinflammatory disease being one extreme of this. There is accumulating evidence that polygenic conditions, such as IRP, sit under the umbrella of autoinflammatory disease. This is based on clinical and genetic similarities between IRP and monogenic disorders. Additionally, interleukin-1, a key innate immune system mediator has been identified as fundamental in IRP pathogenesis, demonstrated by the effectiveness of interleukin-1 blockade in preventing further IRP disease flares.

• The differential for pericarditis is broad and includes infective causes such as certain viruses, bacteria or tuberculosis, autoimmune causes, malignancy, and autoinflammatory disease. To make a diagnosis of IRP alternative causes need to be ruled out sufficiently. While IRP does present predominantly with pericarditis, systemic inflammation and extrapericardial effusions are other key features. Pericarditis can be a feature of monogenic autoinflammatory disease, but is not usually the predominant complaint.

• There is significant morbidity associated with use of corticosteroids in IRP that is refractory to initial management with colchicine and NSAIDs. In these patients, targeted therapy, such as anakinra, should be considered and may be used in preference to corticosteroids. This case highlights that the use of anakinra can result in complete and sustained disease remission. This fuels the case that there is a need for broader access to anakinra for treatment of IRP. Further research is needed to fully understand the pathogenesis of IRP, with the hope that this would help identify additional treatment options to a historically difficult-to-treat, debilitating disease.

